# Global, regional, and national burden of lip and oral cavity cancer and projections to 2036

**DOI:** 10.1186/s12885-025-14995-z

**Published:** 2025-10-14

**Authors:** Shanshan Meng, Anna Lv, Na Li, Xiuna Ding

**Affiliations:** Qingdao Stomatological Hospital Affiliated to Qingdao University, Qingdao, Shandong Province 266001 China

**Keywords:** Lip and oral cavity cancer, Global burden of disease study, Incidence, Mortality, Disability-adjusted life-years, Risk factors, Prediction, Global health

## Abstract

**Background:**

Lip and oral cavity cancer (LOCC) constitutes a significant global health burden with substantial impact on cancer-related morbidity and mortality. Despite therapeutic advances and enhanced preventive strategies, marked disparities in LOCC outcomes persist across populations. This study aims to present a comprehensive temporal analysis utilizing the Global Burden of Disease Study (GBD) 2021, while providing projections through 2036 and examining the contributory effects of principal risk factors.

**Methods:**

Leveraging GBD 2021 data, we analyzed temporal trends in LOCC incidence, deaths, and disability-adjusted life-years (DALYs) across global, regional, and national scales from 1990 to 2021. Future projections through 2036 were generated using the Bayesian Age-Period-Cohort (BAPC) model. We quantified the attributable burden associated with primary risk factors—smoking, alcohol use, and chewing tobacco—stratifying analyses by sex, geographical region, and socio-demographic index (SDI).

**Results:**

Globally, 421,577 incident LOCC cases were documented in 2021, with South Asia demonstrating the highest burden (age-standardized rate [ASR], 9.8 per 100,000). Mortality increased by 113.9% since 1990, reaching 208,379 deaths in 2021. LOCC-associated DALYs totaled 5,874,070 years, with disproportionate impact on low and low-middle SDI regions. Risk factor attribution analysis revealed that 23.4%, 19.2%, and 18.7% of mortality was associated with smoking, alcohol use, and chewing tobacco, respectively. Projections indicate declining mortality trends but sustained increases in incidence and DALYs, particularly in females.

**Conclusions:**

These findings illuminate the escalating global burden of LOCC, with pronounced impact in low and low-middle SDI regions, particularly South Asia. The observed trends emphasize the imperative for implementing targeted prevention strategies, enhancing early detection programs, and establishing comprehensive policies addressing modifiable risk factors to mitigate the projected burden in high-risk populations.

**Supplementary Information:**

The online version contains supplementary material available at 10.1186/s12885-025-14995-z.

## Introduction

Lip and oral cavity cancer (LOCC), a distinctive subset within the broader spectrum of head and neck malignancies, represents a formidable global health challenge, characterized by substantial contributions to cancer-related morbidity and mortality. Recent epidemiological data from 2024 documented approximately 58,450 incident cases and 12,230 fatalities worldwide, underscoring an escalating burden particularly pronounced in regions with prevalent tobacco, alcohol, and betel quid consumption [1, 2]. Epidemiological investigations have consistently demonstrated that tobacco use and alcohol consumption constitute predominant etiological factors, accounting for a disproportionate percentage of the global disease burden, notably in low income countries where survival metrics remain suboptimal [3]. While advancements in early detection methodologies and preventive interventions, including targeted public health initiatives addressing modifiable risk factors, have yielded improved outcomes in certain regions, significant disparities persist, necessitating strategically focused regional interventions [4]. Furthermore, the complex interplay of demographic and socioeconomic determinants significantly influences both incidence and mortality patterns, emphasizing the imperative for comprehensive strategies tailored to these high-risk populations.

The etiology of LOCC is well-established, demonstrating distinct variations across geographical and demographic strata. Tobacco consumption, chronic alcohol use, and betel quid mastication emerge as primary contributory factors, often compounded by limited healthcare accessibility and educational disparities within affected populations [5, 6]. Contemporary research validates that tobacco and alcohol synergistically amplify LOCC risk through the promotion of carcinogenic processes in oral tissues, with betel nut consumption introducing an additional significant risk factor, particularly prevalent in Asian populations [7]. Recent scientific discourse has increasingly focused on the role of human papillomavirus (HPV) in LOCC pathogenesis, especially within developed nations, although its etiological significance remains less pronounced compared to other head and neck malignancies [8]. Regional cultural practices, such as tobacco chewing endemic to South Asia and Mediterranean regions, introduce additional complexity to disease management paradigms. Addressing these multifaceted disparities necessitates the implementation of region-specific health initiatives that comprehensively consider indigenous practices and socioeconomic determinants in mitigating underlying behavioral risk factors. In recent years, rapid technological innovations—particularly the integration of the Internet of Things (IoT) into healthcare—have begun to reshape cancer diagnostics and care pathways. The Internet of Medical Things (IoMT) enables real-time monitoring, remote diagnostics, and AI-driven decision support systems. In the context of LOCC, IoMT has shown promise in enhancing early detection through sensor-enabled screening, monitoring of lesion progression, and facilitating patient-centered follow-up care [9].

Despite extensive knowledge of etiological factors, progress in reducing LOCC incidence and mortality has been modest. Survival metrics demonstrate marked geographical variation, with higher-income nations consistently exhibiting superior outcomes compared to lower-income counterparts [10]. This disparity largely stems from inequities in healthcare infrastructure, early detection capabilities, and accessibility to advanced therapeutic modalities. Consequently, comprehensive understanding of LOCC epidemiological trends becomes paramount for identifying high-burden regions, vulnerable populations, and emerging disease patterns. Moreover, rigorous examination of these trends can inform evidence-based policy formulation aimed at mitigating disease impact, particularly within regions demonstrating the highest disease burden.

The Global Burden of Disease Study (GBD) 2021 provides an unparalleled, comprehensive epidemiological database encompassing incidence, deaths, and disability-adjusted life years (DALYs) across diverse health conditions [11]. Through its provision of temporally and geographically comparable data, the GBD 2021 facilitates nuanced understanding of LOCC burden evolution. Utilizing the most recent GBD 2021 data, this investigation aims to conduct a comprehensive analysis of LOCC trends from 1990 to 2021, examining patterns at global, regional, and national levels. The analysis specifically focuses on incidence, mortality, and DALYs to elucidate both historical and contemporary trends in disease burden. This study encompasses three primary objectives: delineation of temporal disease burden patterns, identification of regions and populations bearing disproportionate disease burden, and exploration of potential correlations with socio-demographic and lifestyle determinants. Through analysis of this extensive temporal dataset, this investigation seeks to provide crucial insights into the epidemiological landscape of LOCC.

In conclusion, this comprehensive study leverages robust GBD 2021 data to meticulously map the global, regional, and national burden of LOCC from 1990 to 2021. Furthermore, the analysis extends beyond current epidemiological parameters through sophisticated projections to 2036, offering an integrated perspective encompassing both contemporary and future trends. Through the provision of detailed temporal and spatial analyses, this research aims to substantively contribute to the expanding body of knowledge surrounding LOCC while supporting strategic initiatives designed to ameliorate the substantial health and economic burden associated with LOCC.

## Materials and methods

### Study data

Data for this cross-sectional investigation were derived from the GBD 2021, administered by the Institute for Health Metrics and Evaluation (IHME) (https://vizhub.healthdata.org/gbd-results). The GBD 2021 framework provides extensive epidemiological data encompassing global, regional, and national disease burdens, injuries, and risk factors across 204 countries and territories. For regional analyses, we adopted the GBD-defined seven super regions, which group countries by geographic proximity and epidemiological similarity. These include: (1) High-income; (2) Central Europe, Eastern Europe, and Central Asia; (3) Latin America and Caribbean; (4) North Africa and Middle East; (5) South Asia; (6) Southeast Asia, East Asia, and Oceania; and (7) Sub-Saharan Africa. These super-regional classifications are used by the GBD to facilitate meaningful comparisons and to reflect shared demographic and health system characteristics across groups of countries. This comprehensive repository includes detailed temporal estimates of incidence, deaths, prevalence, and DALYs from 1990 to 2021.

In accordance with GBD classification protocols, LOCC encompasses malignancies originating in the Lips, oral cavity, and associated oral structures, corresponding to International Classification of Diseases 10th edition codes C00-C08 [12]. The GBD 2021 methodology incorporates multiple data streams, including cancer registries, vital registration systems, health surveys, and peer-reviewed literature. Registry data undergo systematic adjustment to account for underreporting, misclassification, and potential systematic biases. Mortality estimates are generated using the Cause of Death Ensemble model (CODEm), a modeling framework that incorporates a range of predictive covariates and multiple model classes to improve robustness [13]. Incidence estimates are derived using DisMod-MR 2.1, a Bayesian meta-regression tool designed to ensure internal consistency across epidemiological parameters [14]. Additionally, spatiotemporal Gaussian process regression (ST-GPR) is employed to smooth estimates over time and geography when direct data are sparse [15]. The current estimates adhere to methodological protocols outlined in recent GBD publications [16, 17].

In addition to incidence and mortality, this study employed DALYs as a comprehensive metric for quantifying disease burden. This composite DALY metric enables systematic burden comparisons across geographical regions and temporal intervals. Additionally, this study assessed mortality and DALYs associations with established risk factors—smoking, alcohol use, and chewing tobacco—selected in accordance with World Cancer Research Fund guidelines. Age-standardized incidence, mortality, and DALY rates were calculated using the GBD world standard population, which is a synthetic population structure developed by the Institute for Health Metrics and Evaluation (IHME) to allow comparable cross-country and temporal analyses [18]. This standardization process eliminates confounding effects of demographic variations across regions and time periods. All data were accessed through the publicly available GBD 2021 database. Given the aggregate nature of the data, individual informed consent was not required. The investigation adhered to the strengthening the reporting of cohort, cross-sectional, and case-control studies in surgery (STROCSS) guidelines [19].

### Statistical analysis

The analytical framework for this study encompassed comprehensive statistical approaches to examine temporal trends, patterns, and projections in LOCC. ASRs per 100,000 population were computed for incidence, mortality, and DALYs to assess changes in disease burden over time. Temporal trends were analyzed through multiple methodologies, including joinpoint regression analysis for annual percentage change (APC) average annual percentage change (AAPC) determination [20]. The joinpoint regression parameters were specified as follows: minimum of 4 observations between two joinpoints, minimum of 3 observations from a joinpoint to data endpoints, maximum of 5 joinpoints, with model selection based on the Bayesian Information Criterion (BIC) at a significance level of 0.05. We computed the AAPC as a weighted average of the APCs, expressed as AAPC = (e^∑wiβi/∑wi^ − 1) × 100, where wi represents the length of each segment over the total interval, and βi denotes the slope coefficient for each segment.

The formula of the lower and upper confidence interval were as follows:

$$AAPC_{lower\left(a\right)}\;=\left\{\exp\left[\mathrm{ln}(({\textstyle\frac{AAPC}{100}})+1-{\mathrm Z}_{1\;-\;\frac{\mathrm a}2}\sqrt{{\textstyle\sum_{}}\widetilde{\mathrm\omega}_{\mathrm i}^2\mathrm\sigma_{\mathrm i}^2}\right]-1\right\}$$

$$AAPC_{upper\left(a\right)}\;=\left\{\exp\left[\mathrm{ln}(({\textstyle\frac{AAPC}{100}})+1+{\mathrm Z}_{1\;-\;\frac{\mathrm a}2}\sqrt{{\textstyle\sum_{}}\widetilde{\mathrm\omega}_{\mathrm i}^2\mathrm\sigma_{\mathrm i}^2}\right]-1\right\}$$

Temporal trends in ASRs of LOCC were quantified using the estimated annual percentage change (EAPC), derived from a log-linear regression model: EAPC = (e^β^−1) × 100, where β represents the regression coefficient [21]. Trend significance was determined through 95% CIs, with trends considered significant when CIs excluded zero. To characterize geographical patterns, hierarchical cluster analysis stratified 204 regions into four distinct categories based on temporal trajectories and etiological factors, enabling identification of regions with similar disease burden evolution and risk factor profiles. The Bayesian Age-Period-Cohort (BAPC) model was applied to analyze and project trends through 2036, with detailed information on this method available in earlier publications [22, 23]. This sophisticated modeling approach integrated historical data patterns with probability distributions, accounted for age, period, and cohort effects, and generated sex-specific and age-group-specific projections, enabling comprehensive trend analysis across demographic subgroups.

A systematic decomposition analysis was performed to partition changes in disease burden into contributions from population growth, population aging, and changes in age-specific incidence and mortality rates, following the standard decomposition framework used in GBD comparative risk assessment studies [24]. The 95% uncertainty intervals (UIs) were computed using 1,000 random draws from the posterior distribution generated by the GBD modeling process. The 2.5th and 97.5th percentiles of the ordered draws were reported as the lower and upper bounds of the 95% UIs, respectively [25].

Statistical significance was determined through UIs overlap assessment, with comparisons between regions, sexes, or temporal periods considered significant when UIs did not overlap. All statistical analyses were conducted using R (v4.3.2) and Joinpoint (v5.1.0) software. Statistical significance was consistently defined at *P* < 0.05. Results are presented with corresponding 95% UIs to reflect the precision of estimates and facilitate appropriate interpretation of findings.

## Results

### Global LOCC burden

**Fig. 1:**
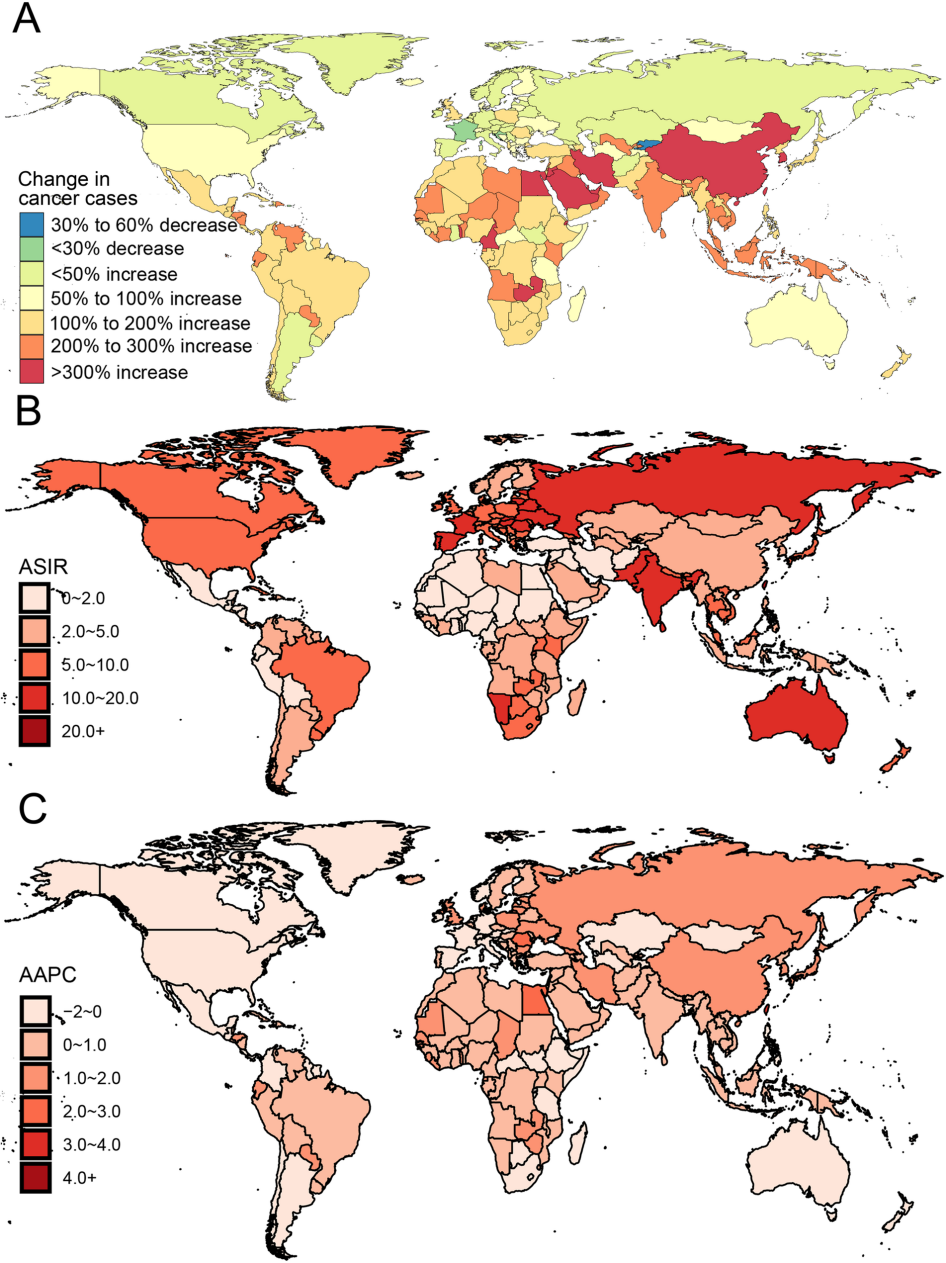
Geographic distribution and temporal trends of LOCC burden across 204 countries and territories. **A** Change in incident cases from 1990 to 2021; (**B**) ASIR per 100,000 population in 2021; (**C**) AAPC of incidence from 1990 to 2021. ASIR, Age-standardized incidence rate; AAPC, Average annual percentage change

Based on GBD 2021 estimates, global incidence of LOCC reached 421,577 new cases in 2021, with predominant concentration in South Asia. The global age-standardized incidence rate (ASIR) was 4.9 per 100,000 population, demonstrating substantial geographical variation ranging from 0.2 in Sao Tome and Principe to 26.5 in Palau. Continental analysis revealed South Asia exhibiting the highest ASIR (9.8) contrasting with Western Sub-Saharan Africa’s lowest (1.3). Between 1990 and 2021, global LOCC incidence demonstrated a 142.2% increase, escalating from 174,077 to 421,577 cases. Notable gender disparity was evident, with male ASIR (6.7) exceeding female ASIR (3.3) by a factor of approximately 2.0. Figure 1B illustrates the geographical distribution of ASIR across 204 countries and territories.

Global LOCC mortality reached 208,379 deaths in 2021, representing a 113.9% increase from 97,420 deaths in 1990. The global age-standardized mortality rate (ASMR) was 2.4 per 100,000 population, ranging from 0.1 (Sao Tome and Principe) to 15.5 (Palau). South Asia demonstrated the highest continental ASMR (6.5), while North Africa and the Middle East exhibited the lowest (0.7). Gender-specific analysis revealed male ASMR (3.4) approximately doubling female ASMR (1.6). SDI stratification indicated elevated mortality rates in low to low-middle SDI regions, underscoring disparities in cancer prevention and treatment capacity.

In 2021, the global LOCC burden accounted for 5,874,070 DALYs, with South Asia bearing the highest regional burden. The global age-standardized DALYs rate (ASDR) in 2021 was 67.7 per 100,000 population, ranging from 2.5 (Sao Tome and Principe) to 432.7 (Palau). This represents a modest 2.3% increase since 1990, reflecting relatively stable DALY rates despite rising absolute case counts, likely due to improvements in cancer management and survival. South Asia recorded the highest continental ASDR (182.3) in 2021, contrasting with North Africa and the Middle East (14.8). Male ASDR (94.6) in 2021 exceeded female ASDR (42.6) by 2.2-fold. Table 1 provides comprehensive epidemiological metrics. 

**Table 1:**
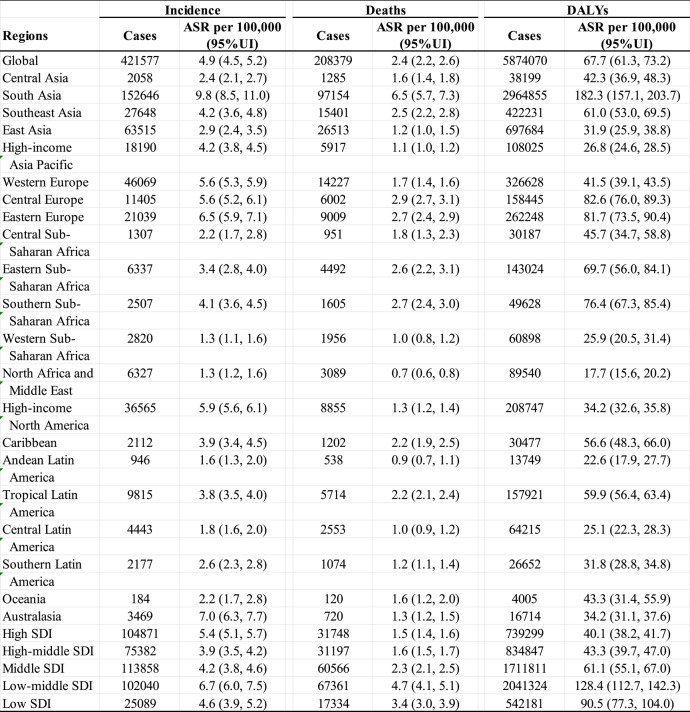
Global and regional burden of LOCC in 2021: incidence, deaths, and DALYs with corresponding ASR stratified by GBD regions

*ASR *Age Standardized Rate, *DALYs* Disability-Adjusted Life Years, *SDI* Soci-Demographic Index

## Trends in global burden of LOCC

**Fig. 2:**
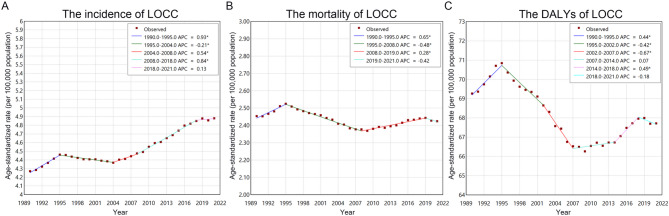
Temporal trend analysis of LOCC burden using joinpoint regression: annual percent change (APC) and average annual percent change (AAPC) in age-standardized rates for (**A**) incidence, (**B**) mortality, and (**C**) disability-adjusted life years (DALYs)

This study evaluated temporal changes in the burden of LOCC across 204 countries and territories from 1990 to 2021, focusing on trends in incidence, mortality, and DALYs. Changes in LOCC cases were categorized into ranges of percentage increase or decrease and visualized in Fig. 1A. To further explore incidence trends, we calculated the Average Annual Percent Change (AAPC) and visualized the results in Fig. 1C. The AAPC analysis identified countries with the most pronounced decreases in incidence, including Kuwait (AAPC: −2.8), Kyrgyzstan (AAPC: −2.5), and Puerto Rico (AAPC: −2.0). Conversely, substantial increases in incidence were observed in regions such as Cabo Verde (AAPC: 11.6), Taiwan (Province of China) (AAPC: 3.4), and the Northern Mariana Islands (AAPC: 2.8), as detailed in Table S1 and S2. At the continental level, global trends showed an overall increase in LOCC incidence for men (AAPC: 0.3) and women (AAPC: 0.8). Among men, Cabo Verde exhibited the most rapid increase in incidence (AAPC: 11.0), whereas Afghanistan displayed the slowest growth (AAPC < 0.1). Globally, the ASIR demonstrated a steady upward trajectory from 2004, as shown in Fig. 2.

Mortality trends varied by sex, with women exhibiting an increasing rate of deaths (AAPC: 0.3), while men experienced a decline (AAPC: −0.2). Significant reductions in mortality were recorded in Kuwait (AAPC: −3.3), Kyrgyzstan (AAPC: −2.8), and Puerto Rico (AAPC: −2.9), likely reflecting effective risk factor control and improvements in early detection. In contrast, regions such as Cabo Verde (AAPC: 11.1), Northern Mariana Islands (AAPC: 2.7), and Taiwan (Province of China) (AAPC: 2.5) showed marked increases in mortality. On a continental scale, Western Sub-Saharan Africa exhibited the greatest rise in mortality (AAPC: 0.6), consistent with rising incidence and limited healthcare infrastructure, while Central Europe experienced the smallest increase (AAPC: 0.1). When analyzed by SDI, low (AAPC: 0.3) and low-middle SDI regions (AAPC: 0.3) showed the most rapid increases in mortality, as outlined in Table S3 and S4.

In terms of DALYs, a decreasing trend was observed globally among men (AAPC: −0.2), whereas women exhibited an increasing trend (AAPC: 0.2) (Figure S1). The greatest reductions in DALYs occurred in Kuwait (AAPC: −3.8), Kyrgyzstan (AAPC: −3.1), and Puerto Rico (AAPC: −2.7). Conversely, Cabo Verde (AAPC: 10.9), Northern Mariana Islands (AAPC: 2.6), and Taiwan (Province of China) (AAPC: 2.5) exhibited the highest increases in DALYs. Regionally, Western Sub-Saharan Africa demonstrated the most rapid rise in DALYs (AAPC: 0.5), whereas Central Europe showed the smallest increase (AAPC < 0.1). By SDI classification, low-middle (AAPC: 0.2) and low SDI regions (AAPC: 0.1) experienced the greatest growth in DALYs, emphasizing the ongoing burden in resource-limited settings. Overall, global trends shown an stable burden of DALYs, as summarized in Table S5 and S6.

**Fig. 3:**
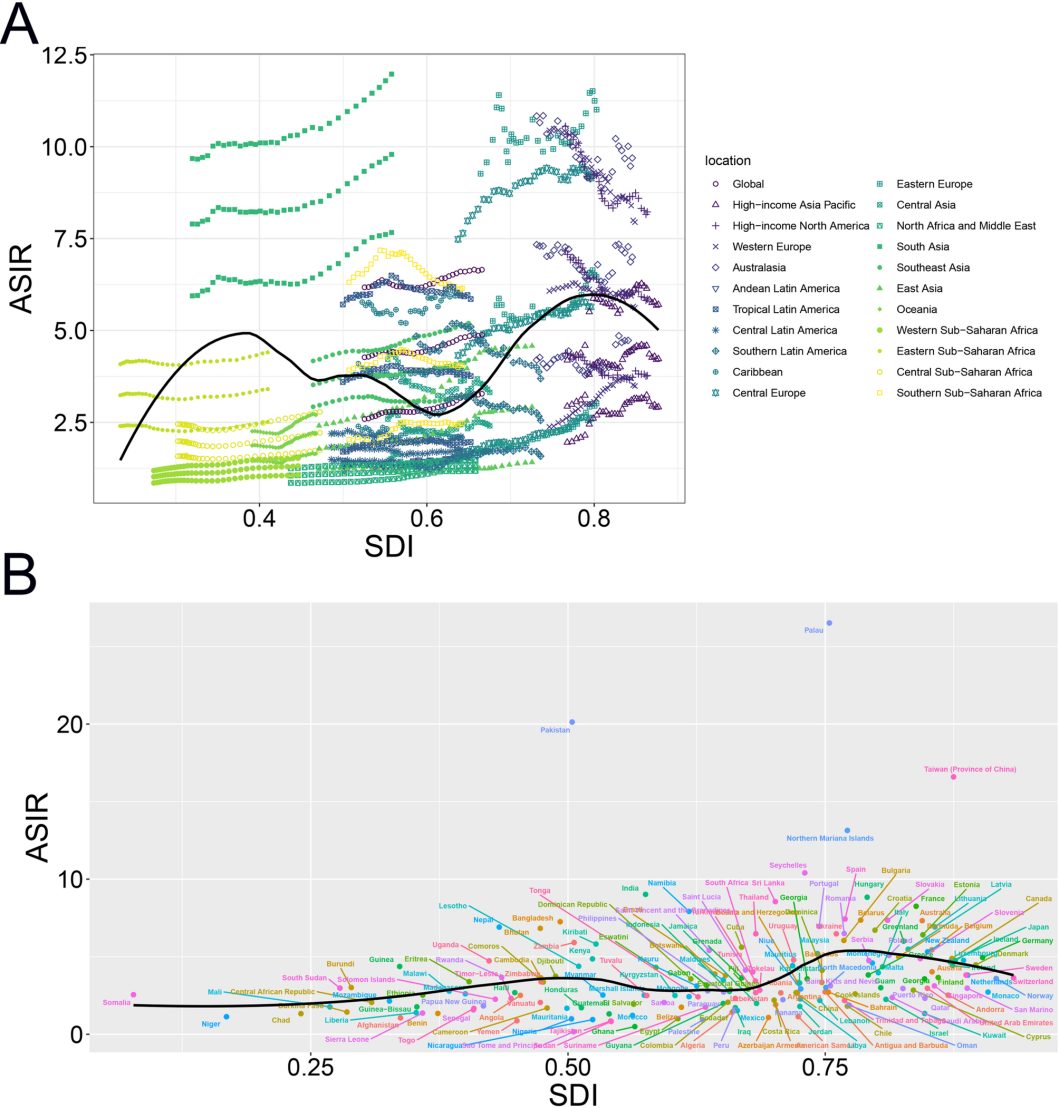
Socio-demographic disparities in LOCC burden: age-standardized incidence rates (ASIR) stratified by socio-demographic Index (SDI) for (**A**) 21 GBD regions and (**B**) 204 countries and territories, 1990–2021

A hierarchical cluster analysis of ASRs for LOCC across 204 countries and territories revealed four distinct trend categories: significant increase, minor increase, stable or minor decrease, and significant decrease, each represented by specific color codes (Figure S2). When analyzing ASRs relative to SDI, regional and national ASRs were compared to expected levels based on SDI, as depicted in Figs. 3 and S3. In 2021, countries with SDI values around 0.4 and 0.8 displayed higher LOCC incidence rates. The relationship between ASIR and SDI was characterized by an initial increase, peaking in South Asia, followed by a decline with higher SDI levels (Fig. 3A). A similar trend was observed in mortality, where ASMR peaked in Eastern Europe before declining as SDI values rose, as shown in Figure S3, reflecting the combined influence of risk factor prevalence, healthcare access, and demographic transitions.

### LOCC burden by risk factors

**Fig. 4:**
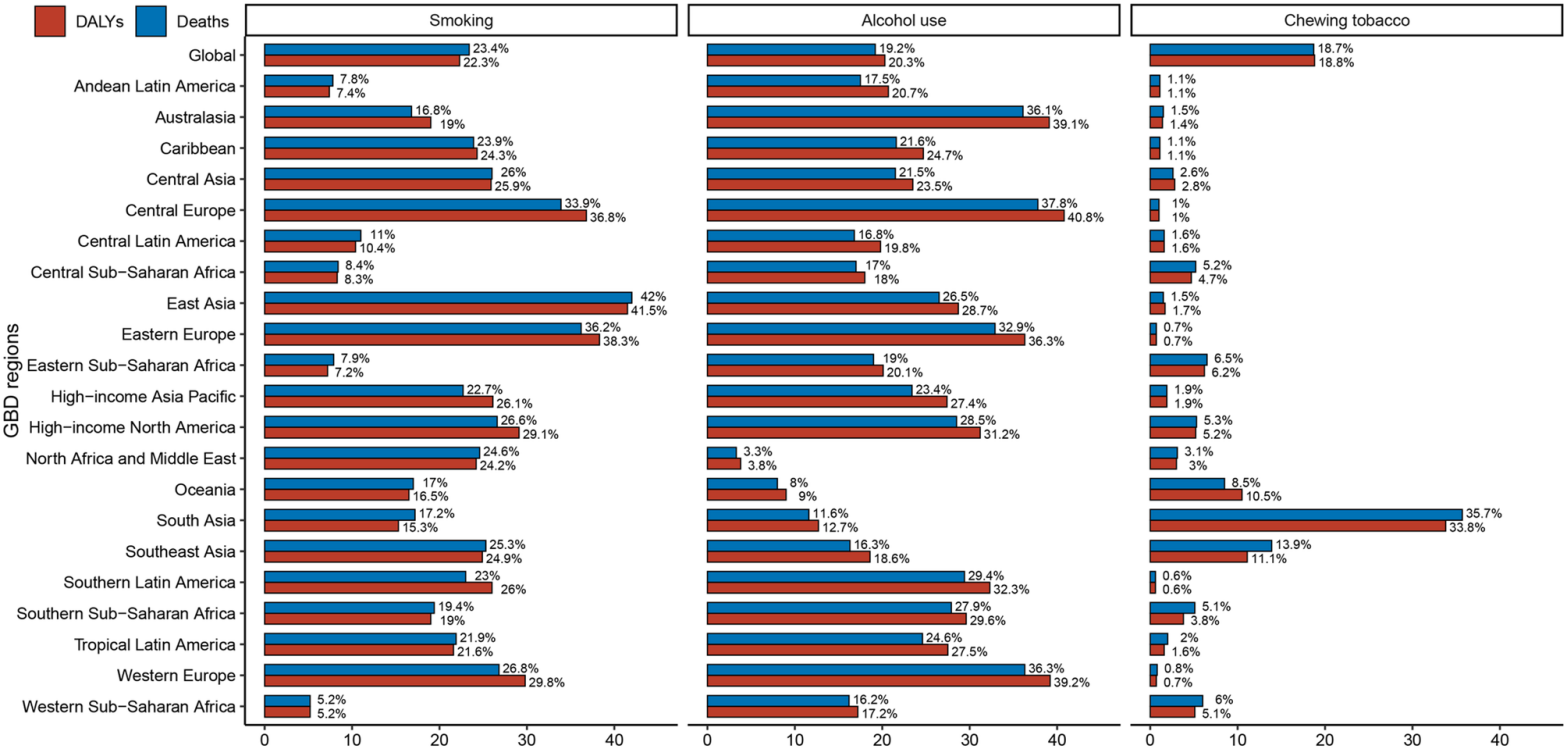
Risk factor attribution analysis for LOCC in 2021: proportional contribution of smoking, alcohol use, and chewing tobacco to deaths and disability-adjusted life years (DALYs) across 21 GBD regions

A substantial proportion of global LOCC mortality and DALYs was attributable to three primary risk factors based on GBD 2021 estimates. Smoking accounted for 23.4% of global deaths (95% UI: 16.4–30.0), followed by alcohol use (19.2%; 95% UI: 15.0–23.3) and chewing tobacco (18.7%; 95% UI: 14.2–22.7). Similarly, smoking contributed to 22.3% of DALYs (95% UI: 15.7–28.4), alcohol use to 20.3% (95% UI: 15.8–24.5), and chewing tobacco to 18.8% (95% UI: 14.2–23.0). The burden of these risk factors varied significantly by region. In East Asia, smoking was the predominant contributor to mortality (42%; 95% UI: 31.0–51.5) and DALYs (41.5%; 95% UI: 30.8–50.7). Central Europe had the highest burden attributable to alcohol use for both deaths (37.8%; 95% UI: 30.6–44.4) and DALYs (40.8%; 95% UI: 33.3–47.3). In South Asia, chewing tobacco was the leading contributor to mortality (35.7%; 95% UI: 27.9–44.0) and DALYs (33.8%; 95% UI: 26.0–41.5), as depicted in Fig. 4.

## Trends of LOCC from 1990 to 2036

**Fig. 5:**
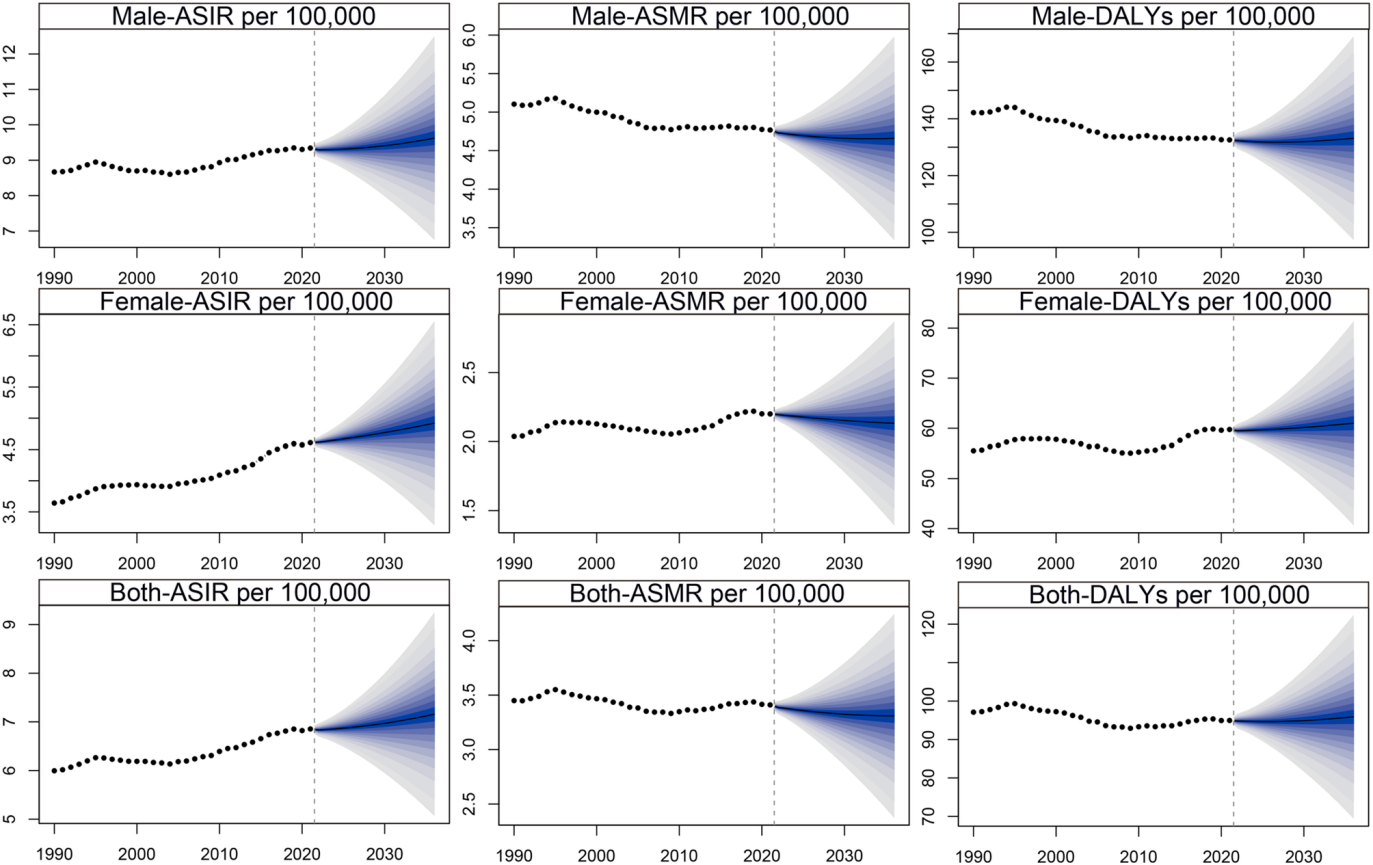
Bayesian age-period-cohort (BAPC) model projections of LOCC burden by gender, 1990–2036: projected trends in age-standardized incidence rate (ASIR), age-standardized mortality rate (ASMR), and age-standardized DALY rate

The trends in LOCC burden from 1990 to 2036 revealed significant fluctuations (Fig. 5). The ASIR for both sexes peaked in 1995, followed by a decline until 2004. Subsequently, ASIR exhibited a steady increase for both males and females. Mortality trends differed by sex: ASMR for males declined steadily from 1995 to 2019, while females experienced a decline until 2008, followed by an increase peaking in 2019. Post-2019, ASMR for both sexes showed a consistent decline. For DALYs, trends exhibited notable fluctuations between 1990 and 2036. Recent years, the ASRs of DALYs peaked in 2019, followed by a decline until 2027, after which it resumed an upward trajectory. For males, DALY ASRs steadily decreased from 1994 to 2028, while females showed more variable patterns.

## Discussions

Our comprehensive analysis of the GBD 2021 data elucidates significant temporal and spatial patterns in the global burden of LOCC from 1990 to 2021, with projections extending to 2036. The study revealed substantial disease burden metrics, with 421,577 incident cases, 208,379 deaths, and 5,874,070 DALYs documented in 2021. These findings demonstrate the persistent public health challenge posed by LOCC, characterized by marked heterogeneity across geographical regions and demographic groups. The observed longitudinal increases in LOCC incidence, mortality, and DALYs since 1990 reflect complex interactions between demographic transitions, evolving lifestyle patterns, and the expanding prevalence of established risk factors, including tobacco consumption, alcohol use, and smoking.

A salient finding emerges in the marked geographical heterogeneity of LOCC incidence. South Asia demonstrated the highest burden of new cases, followed by Eastern Europe, while Western Sub-Saharan Africa reported substantially lower incidence rates. The ASIR in South Asia (9.8) exceeded that of Western Sub-Saharan Africa (1.3) by nearly sevenfold. This pronounced disparity likely reflects multiple factors, including differential exposure to risk factors—particularly the prevalent practice of tobacco chewing in South Asia—and disparities in healthcare accessibility and early detection capabilities. The notably lower incidence rates in Western Sub-Saharan Africa may partially reflect limitations in diagnostic infrastructure and surveillance systems, potentially resulting in case underascertainment. Furthermore, significant sex-specific disparities were observed globally, with males exhibiting higher incidence rates (ASIR: 6.7) compared to females (ASIR: 3.3). This sexual dimorphism in LOCC incidence aligns with established patterns of risk factor exposure, particularly regarding tobacco and alcohol consumption, and may be further influenced by occupational exposures and healthcare utilization patterns [26].

Mortality patterns demonstrated significant regional variation, with South Asia bearing a disproportionate burden of LOCC-related deaths, concordant with its elevated incidence rates and consistent with previous epidemiological investigations [27, 28]. In contrast, North Africa and the Middle East exhibited markedly lower mortality rates, potentially attributable to variations in healthcare infrastructure, early detection protocols, and therapeutic interventions [29]. The relatively lower mortality burden in these regions may also reflect distinct patterns of tobacco consumption, characterized by lower prevalence rates compared to global averages [30]. Notably, certain geographical areas, including Cabo Verde and the Northern Mariana Islands, demonstrated concerning upward trajectories in mortality rates, highlighting emergent challenges in regions with limited oncological infrastructure. Conversely, nations such as Kuwait and Kyrgyzstan demonstrated sustained decreases in mortality, underscoring the potential impact of strengthened healthcare systems on disease outcomes.

The longitudinal analysis of global DALY trends revealed complex temporal patterns from 1990 to 2036, with notable inflection points. DALYs, as a composite metric incorporating both years of life lost due to premature mortality and years lived with disability, provide crucial insights into the comprehensive societal impact of LOCC [31]. Our projections indicate that the age-standardized DALY rate reached its apex in 2019 and is projected to demonstrate a declining trajectory through 2027, followed by a gradual resurgence. This pattern suggests that while mortality rates may show improvement, the chronic sequelae of LOCC—including treatment-related morbidity and metastatic complications—will continue to pose significant challenges to healthcare systems globally. The geographical distribution of DALYs exhibited substantial heterogeneity, reflecting both disease incidence patterns and healthcare system capabilities. South Asia’s elevated DALY burden corresponded with its high incidence rates, while regions such as Central Europe demonstrated more favorable DALY profiles, likely attributable to robust healthcare infrastructure and effective early detection programs [32].

A critical finding emerging from our analysis is the substantial attributable burden associated with modifiable risk factors, particularly smoking, alcohol use, and chewing tobacco. Our analysis indicates that smoking was the predominant risk factor, accounting for 23.4% of mortality and 22.3% of DALYs globally. Alcohol consumption and chewing tobacco demonstrated comparable contributions, responsible for 19.2% and 18.7% of deaths, and 20.3% and 18.8% of DALYs, respectively. These findings underscore the imperative for targeted public health interventions aimed at risk factor modification, particularly in high-burden regions. The relative contribution of these risk factors demonstrated marked regional variation. East Asia exhibited the highest smoking-attributable burden, with approximately 42.0% of both deaths and DALYs attributable to this risk factor, reflecting the persistent high smoking prevalence in countries such as China and Japan despite ongoing tobacco control initiatives [33]. Central Europe demonstrated a distinct pattern, with alcohol consumption emerging as the dominant risk factor, accounting for 37.8% of mortality and 40.8% of DALYs, suggesting strong sociocultural determinants of alcohol-related LOCC burden in this region [34]. South Asia’s unique risk factor profile was characterized by the substantial burden attributable to smokeless tobacco, contributing to 35.7% of deaths and 33.8% of DALYs, highlighting the critical need for region-specific interventions targeting culturally embedded tobacco use practices.

Despite increasing recognition of the importance of early detection and preventive strategies, significant challenges persist in mitigating the global LOCC burden. The observed disparities in disease metrics across regions reflect both inequitable distribution of healthcare resources and persistent exposure to established risk factors. Regions such as South Asia, characterized by high incidence and mortality rates, require comprehensive public health strategies encompassing prevention, early detection, and therapeutic interventions [35]. While tobacco control policies have demonstrated efficacy in certain contexts, additional targeted interventions are necessary to address the high prevalence of chewing tobacco in South Asia [36]. In regions reporting lower disease burden, such as Western Sub-Saharan Africa, strengthening surveillance infrastructure and cancer registry systems is paramount for accurate burden estimation. The current limitations in cancer registration systems in many low and low-middle income countries potentially result in burden underestimation and delayed intervention implementation. Enhancement of cancer registry infrastructure and diagnostic capabilities represents a critical priority for improved disease monitoring and outcomes optimization [37].

The increasing global burden of LOCC, particularly in low- and middle-SDI regions, underscores the urgent need for targeted public health interventions. These findings highlight the importance of scaling up preventive strategies such as anti-tobacco and alcohol reduction campaigns, early detection programs, and public awareness initiatives. Moreover, countries with high or rising AAPCs should consider integrating LOCC surveillance into broader cancer control plans to ensure timely diagnosis and equitable access to treatment. Policymakers can also use regional ASMR and ASDR trends to guide resource allocation and prioritize interventions tailored to the specific epidemiological context.

The findings of this study highlight several avenues for future research in LOCC. First, longitudinal cohort and molecular studies are needed to better understand the interaction between traditional risk factors—such as smoking, alcohol use, and chewing tobacco—and emerging factors like HPV infection and environmental exposures. Second, evaluation of the effectiveness of prevention and early detection programs in high-burden regions could provide evidence for scaling up context-specific interventions. Third, integration of novel technologies, including the Internet of Medical Things (IoMT) and AI-assisted screening, warrants exploration to enhance early diagnosis and surveillance in resource-limited settings. Finally, comparative projections using future GBD releases, such as GBD 2023, will be essential for validating these findings and monitoring progress toward global oral cancer control goals.

While our study provides comprehensive insights into the global, regional, and national LOCC burden, several methodological Limitations warrant acknowledgment. Primary among these is the reliance on GBD 2021 estimates, which, while robust, are subject to modeling assumptions that may not fully capture the complexity of LOCC epidemiology in certain contexts. Case under ascertainment, particularly in resource-limited settings with constrained healthcare infrastructure, may result in burden underestimation. Additionally, variations in diagnostic protocols, cancer classification systems, and healthcare accessibility may influence data accuracy. Future research priorities should include strengthening cancer registration systems in underrepresented regions and conducting robust population-based studies to refine burden estimates. Moreover, expanded investigation of genetic and environmental determinants of LOCC, particularly in high-burden regions, will be essential for developing targeted prevention and treatment strategies.

## Conclusions

This comprehensive analysis demonstrates the significant global burden of LOCC, revealing marked regional disparities in incidence, mortality, and DALYs. Our findings identify South Asia, Eastern Europe, and East Asia as high-burden regions, where smoking, alcohol consumption, and chewing tobacco significantly influence disease patterns. Notable sex-specific differences show males experiencing higher incidence rates, reflecting differential exposure to risk factors. Despite improvements in some regions, the global LOCC burden remains a pressing public health challenge, particularly in low and low-middle income countries with limited healthcare infrastructure and cancer surveillance capabilities. Addressing these disparities requires targeted prevention strategies, enhanced early detection programs, and public health interventions tailored to regional risk factors. The development of robust cancer registries, improved data collection systems, and region-specific research are essential for formulating effective strategies to mitigate the growing LOCC burden worldwide.

## Supplementary Information

Supplementary Material 1.

Supplementary Material 2.

Supplementary Material 3.

Supplementary Material 4.

Supplementary Material 5.

## Data Availability

All data used in this study can be downloaded from this link: https://vizhub.healthdata.org/gbd-results/. For additional information, please contact the corresponding author.
