# Supplementary figures and images for: Global, regional, and national burden of lip and oral cavity cancer and projections to 2036
Source: BMC Cancer. 2025 Oct 14;25:1573. doi: 10.1186/s12885-025-14995-z (PMC12522484; doi:10.1186/s12885-025-14995-z)

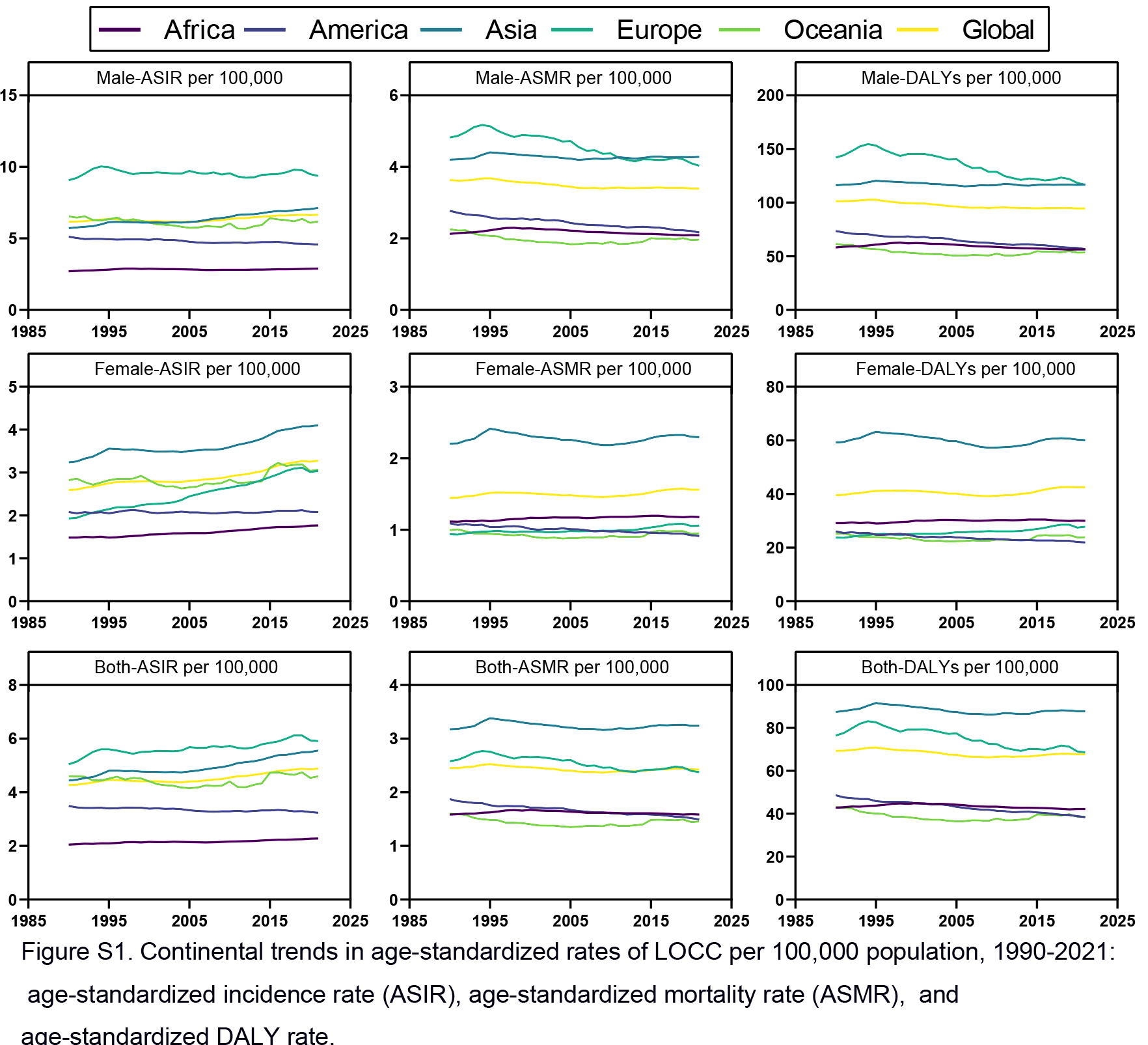

Supplement: Supplementary file 1 — Supplementary Material 1. [file 12885_2025_14995_MOESM1_ESM.tif]

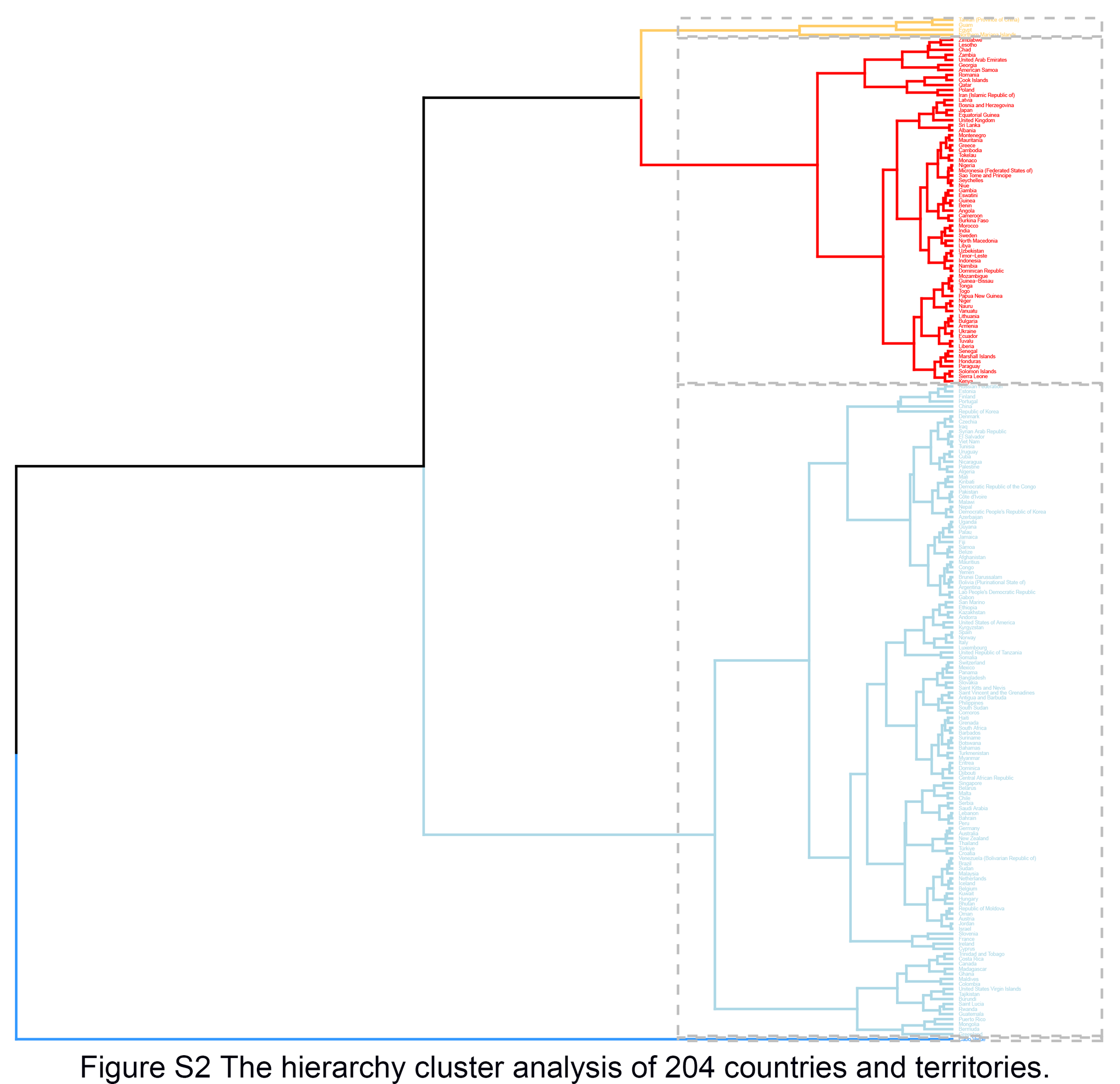

Supplement: Supplementary file 2 — Supplementary Material 2. [file 12885_2025_14995_MOESM2_ESM.tif]

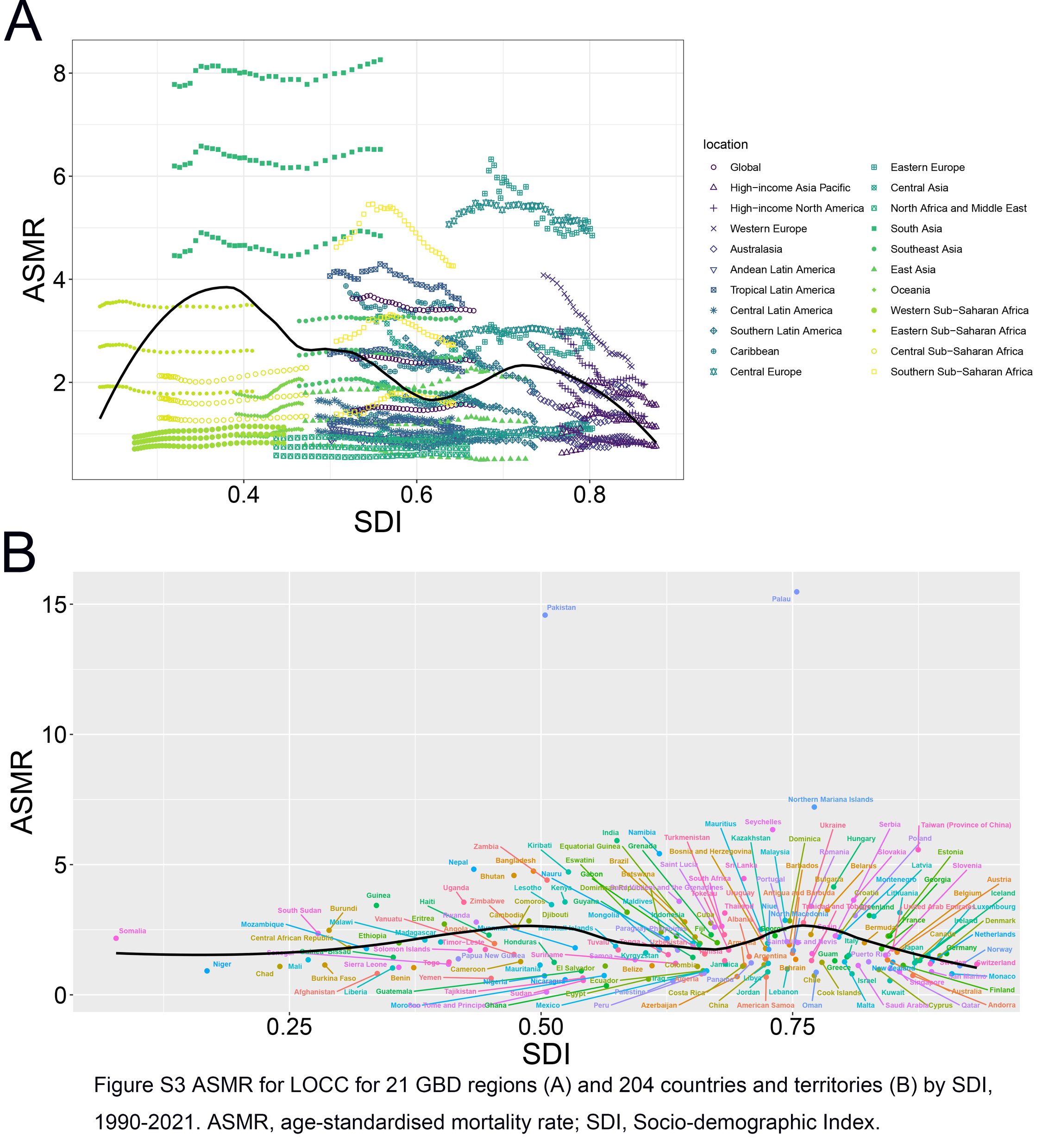

Supplement: Supplementary file 4 — Supplementary Material 4. [file 12885_2025_14995_MOESM4_ESM.tif]
