# Supplementary material for: Global, regional, and national burden of lip and oral cavity cancer and projections to 2036
Source: BMC Cancer. 2025 Oct 14;25:1573. doi: 10.1186/s12885-025-14995-z (PMC12522484; doi:10.1186/s12885-025-14995-z)
Supplement: Supplementary file 5 — Supplementary Material 5. [file 12885_2025_14995_MOESM5_ESM.docx]

**Supplementary Methods**

**1. Estimation of LOCC Burden Using the GBD 2021 Framework**

This study is based on publicly available estimates from the Global Burden of Disease (GBD) Study 2021, coordinated by the Institute for Health Metrics and Evaluation (IHME). The GBD framework systematically estimates cause-specific morbidity and mortality metrics by age, sex, year, and location using a comprehensive modeling strategy that harmonizes disparate data sources across countries and time periods.

**Data Sources:**

The GBD 2021 utilized multiple data sources for lip and oral cavity cancer (LOCC), including cancer registries, vital registration systems, verbal autopsy data, and hospital records. These were mapped to the International Classification of Diseases (ICD-10 codes: C00–C08) to standardize case definitions.

**Cause of Death Modeling:**

Mortality estimates for LOCC were generated using the Cause of Death Ensemble model (CODEm), a highly flexible modeling approach that combines multiple models (e.g., mixed-effects, spatial-temporal Gaussian process regression) into an ensemble framework. CODEm identifies the best-performing predictive models through out-of-sample validation techniques.

**Non-Fatal Estimates:**

The DisMod-MR 2.1 tool, a Bayesian meta-regression modeling platform, was employed to generate consistent estimates of incidence, prevalence, remission, and disability due to LOCC. It ensures internal consistency across epidemiological parameters by integrating various data sources while accounting for biases, sampling errors, and missing data.

**Standardization:**

All rates were age-standardized to the GBD world population using the direct method to ensure comparability across countries and time. Disability-adjusted life years (DALYs) were calculated as the sum of years of life lost (YLLs) due to premature mortality and years lived with disability (YLDs).

**2. Projection Using the Bayesian Age-Period-Cohort (BAPC) Model**

To project LOCC incidence and mortality rates through 2036, we applied a Bayesian Age-Period-Cohort (BAPC) model, a statistical framework that decomposes temporal trends into age, period, and cohort effects while accounting for uncertainty.

**Model Specification:**

The BAPC model assumes a Poisson distribution for the number of incident cases or deaths:

Where is the observed count in age group $a$ and period $p$, is the corresponding rate, and is the population at risk. The log rate is modeled as:

Where μ is the intercept, denotes the age effect, the period effect, and the cohort effect (with $c=p-a$).

**Prior Specification and Computation:**
We assigned second-order random walk (RW2) priors to age, period, and cohort effects to enforce smoothness across adjacent groups. Posterior distributions were estimated using Integrated Nested Laplace Approximation (INLA), implemented in R via the BAPC and INLA packages.

**Projection Window:**
The model was trained on GBD 2021 data from 1990 to 2019, with projections made for the years 2020–2036. Population projections used for forecasting were obtained from the GBD Study.

**Validation:**
We performed out-of-sample validation to evaluate the predictive accuracy of the BAPC model. The model showed good agreement between observed and predicted trends across most regions.
